# 

**Published:** 1909-02-06

**Authors:** 


THE
### GRADUATES' MEDICAL SCHOOLS.

DIARY FOR WEEK FEB. 8 TO FEB. 13.

**NORTH-EAST LONDON POST-GRADUATE COLLEGE, Prince of Wales's General Hospital, Tottenham, N.**

**At 4.30 p.m.**
**Feb. 9**, Dr. Chappell, **Demonstration.**
**Feb. 11**, Mr. J. H. Evans, **Diseases of the Male Breast.**

**HOSPITAL FOR CONSUMPTION AND DISEASES OF THE CHEST, Brompton, S.W.**

**Feb. 10**, Dr. Bosanquet, **Mediastinal Tumours.**

**THE THROAT HOSPITAL, Golden Square, W.**

**At 5.30 p.m.**

**Feb. 8**, Dr. Bond, **Acute and Chronic Tonsillitis.**

**Feb. 11**, Mr. Faulder, **Chronic Pharyngitis, Keratons, &c.**

**NATIONAL HOSPITAL FOR THE PARALYSED AND EPILEPTIC, Queen Sq., Bloomsbury, W.C.**

**At 3.30 p.m.**
**Feb. 9**, Dr. Taylor, **Treatment in Nervous Diseases.**
**Feb. 12**, Dr. Russell, **The Significance of Tremor.**

**THE POST-GRADUATE COLLEGE, West London Hospital, Hammersmith, W.**

**At 10 a.m.**
**Feb. 8 and 11**, Surgical Registrar, **Demonstration.**
**Feb. 12**, Medical Registrar, **Demonstration.**
**At 12 noon.**
**Feb. 8**, Dr. Low, **Pathological Demonstration.**
**At 12.15 p.m.**
**Feb. 10 and 13**, Dr. Pritchard, **Practical Medicine.**
**At 5 p.m.**
**Feb. 8**, Mr. Lloyd Williams, **Diagnosis of Swellings of the Jaws.**
**Feb. 9**, Dr. Seymour Taylor, **Diagnostic Signs in Organic Disease of the Nervous System.—II. The Spinal Cord.**
**Feb. 10**, Dr. Beddard, **Medicine—II.**
**Feb. 11**, Mr. Dunn, **Eye Diseases.**
**Feb. 12**, Dr. Abraham, **Skin Diseases.**

**LONDON SCHOOL OF CLINICAL MEDICINE, Seamen's Hospital, Greenwich, S.E.**

**At 3.30 p.m.**
**Feb. 10**, Mr. Cargill, **Orbital Cellulitis.**
**At 2.30 p.m.**
**Feb. 11**, Dr. Rankin, **Jaundice.**

**ST. JOHN'S HOSPITAL FOR DISEASES OF THE SKIN, Leicester Square, W.C.**

**Feb. 11**, Chesterfield Lectures, **Treatment of Syphilis.**

---

THE HOSPITAL February 6, 1909.

*Name* ......................................................................................

*Address* ......................................................................................

......................................................................................

This Coupon must accompany manuscript or contributions intended for The Hospital.

